# The impact of the use of immunosuppressive treatment after an embryo transfer in increasing the rate of live birth

**DOI:** 10.3389/fmed.2023.1167876

**Published:** 2023-06-27

**Authors:** Mihaela Andreescu

**Affiliations:** ^1^Department of Hematology, Colentina Clinical Hospital, Bucharest, Romania; ^2^Titu Maiorescu University, Bucharest, Romania

**Keywords:** reproductive immunology, Immunosuppressants, recurrent pregnancy loss, immunotherapy, repeated implantation failure

## Abstract

The tolerance of the immune system for the semi-allogeneic embryo is promoted by several factors and the cells involved in the immune system and factors in the mother during pregnancy. The dysregulation of the immune responses between the mother and fetus is a risk factor that raises the likelihood of rejection of the embryo and reproductive failure. To safeguard embryos and prevent immunological attacks, it is critical to suppress immunological rejection and encourage immunological tolerance. Based on current medical literature, it seems that immune cell management through immunosuppressive therapies can address reproductive failures. Immunosuppressive treatment has demonstrated encouraging results in terms of enhancing outcomes related to pregnancy and rates of live birth by regulating the immune responses of mothers and positively impacting the reproductive processes of humans. Currently, there is scarcity of high-quality data regarding the safety and efficacy of immunosuppressive therapies for children and mothers. Therefore, it is important to exercise caution while selecting use of any immunosuppressive therapy in pregnancy. This mini review provides a comprehensive overview of the existing literature regarding the impact of Calcineurin Inhibitors and anti-TNF treatment on improving the live birth rate following embryo transfer.

## Introduction

While having a child is a momentous occasion in life, the ability to conceive without assistance is frequently uncertain. Around 15 % of couples in the global reproductive population experience infertility (1). In recent strides made in scientific research, assisted reproductive technology (ART) has been a major breakthrough for couples who were previously not able to conceive and has also helped in the detection of early miscarriages. However, with the advancement of ART, a new hurdle has come to light, known as recurrent implantation failure (RIF) (2), which poses a challenge for couples trying to achieve a successful pregnancy. Implantation is the process in which the embryo attaches to the luminal surface of the endometrium (3). Successful implantation is indicated by the detection of an intrauterine gestational sac through ultrasonography (4). Negative pregnancy tests and the absence of a visible gestational sac can result from implantation failure during the initial phases of embryo migration or attachment (5). Implantation failure may also occur at a later stage after the embryo has successfully migrated through the endometrial luminal surface and started producing hCG, which can be identified in blood or urine. However, if gestational sac formation is impaired before it can occur, the implantation process will be hindered (5). The implantation rate can be described as the proportion of embryos that result in the production of gestational sacs to the total number of embryos that are transported into the uterine cavity (4).

Studies have shown that the implantation rate during the IVF process is higher when embryos are transferred on day 5 or 6 (40%) compared to day 2 or 3 (25%) (5). Repeated implantation failure (RIF) is a condition that occurs in patients utilizing assisted reproductive techniques where multiple cycles of *in vitro* fertilization and transfer of several high-quality embryos fail to result in a clinical pregnancy (6). Coughlan et al. (5, 7) proposed a commonly accepted definition of RIF which states that the absence of a successful clinical pregnancy in women who are younger than 40 years of age despite a minimum of four good-quality embryos have been transferred in at least three fresh or frozen cycles. The process of implantation is complex and RIF can be influenced by several factors of maternal or embryonic origin. Successful implantation of the embryo, which is considered a homozygous hemizygous antigen, is dependent on various factors (6, 7). For successful embryo synchronization following transfer into the uterine cavity, it is essential that the endometrium is receptive, and that the maternal immune system is able to tolerate the presence of the paternal alloantigen throughout the pregnancy (8). There are several potential factors that may contribute to impaired endometrium receptivity and defective maternal-fetal immunotolerance, including uterine abnormalities, infections, metabolic or hormonal disorders, immunological factors, severe male factors, thrombophilias, or an abnormal immune response. Furthermore, inflammation is believed to have a significant influence on pregnancy and can profoundly affect its outcome (8). While thrombophilia was previously considered as a cause for recurrent abortions when no genetic, local, or infectious causes were identified, recent attention has shifted towards the immunological aspect of pregnancy. Due to this, the need for balancing and immunomodulation has emerged to improve rate of successful pregnancy. Various immunosuppressive drugs have been studied for their potential in this regard. However, ethical considerations limit the scope of such studies to a small patient population. Previously immunosuppressive treatments such as corticosteroids, intravenous immunoglobulin (IVIG), and hydroxychloroquine has shown improved outcomes in some studies (9–11). However, the use of these treatments after an embryo transfer to increase the rate of live birth remains controversial (12). Furthermore, the use of these drugs has been incorporated into IVF protocols in many European Union countries. The recommendations for the utilization of immunosuppressive treatment in clinical practice are based on a relatively low level of evidence which necessitates careful consideration for their use in pregnancy. Methotrexate, mycophenolate, teriflunomide, and mitoxantrone are contraindicated in pregnancy due to their known teratogenic effects. When considering the use of anti-TNF-alpha agents and mTOR inhibitors in the context of pregnancy, it is important to exercise caution as there is limited experience with these drugs in pregnant patients (13). Currently, almost all immunosuppressive and immunomodulatory therapies carry little evidence regarding safety and efficacy for children and mothers. However, these agents have been used in pregnancy for other reasons such as atopic dermatitis which can impart significant clinical benefits (14). Thus, it is essential to gain a deeper understanding of the scientific basis of their use. This mini review aims to provide a comprehensive overview of the current evidence concerning the role of Calcineurin Inhibitors and anti-TNF treatment in enhancing live birth rates.

## Recurrent implantation failure and immune system

The immunological response in pregnancy is a highly intricate and multifaceted process, characterized by the co-existence of both inflammatory and anti-inflammatory mechanisms. The successful outcome of pregnancy hinges on the intricate interplay between these processes. It has been well-established that implantation is marked by a restrained inflammatory state, while mid-pregnancy is marked by a predominance of anti-inflammatory processes. However, as parturition approaches, there is renewed activation of inflammatory pathways (8, 15). Immune cells such as natural killer (NK) cells, dendritic cells, macrophages, and T cells are present in the endometrium and have different functions in regulating endometrial receptivity and facilitating embryo implantation (16). The success of embryo implantation and development is also influenced by immune-related cytokines such as interleukin (IL)-6, IL-10, IL-15, IL-17, interferon gamma (IFN-γ), tumor necrosis factor alpha (TNF-α), and nuclear factor kappa B (NF-κB), which are present in the intima (16, 17). The failure of embryo implantation may be attributed to a decrease in endometrial receptivity, which can result from dysregulation of the endometrial immune profile (2, 5, 18). Studies have reported that a majority of RIF patients, specifically 81.7%, exhibit dysregulation in their endometrial immune profiles. Furthermore, among RIF patients, overactivation of the immune system has been observed in 56.6% of cases while low activation has been observed in 25% (2). Therefore, addressing this mechanism could potentially enhance the chances of successful pregnancies in patients with RIF.

Maternal immune tolerance is induced during the window of implantation (WOI) through a substantial influx of immune cells to shelter the embryo from refusal (19). Maternal immune tolerance during pregnancy is maintained by the delicate interplay of cytokine signals, which is facilitated by immune cells such as T-helper cells (Th1, Th2, and Th17) and regulatory T-cells (Treg) (20). T-helper cells (Th1) play a role in cell-mediated immunity by producing cytokines such as interleukin-2 (IL-2), tumor necrosis factor-alpha (TNF-α), and interferon gamma (IFN-γ), which can trigger inflammation and there is a dominance of Th1 in peri-implantation phase and the regulated TH1 immunity is advantageous for the invading trophoblasts rather than causing harm. On the other hand, Th2 cells are responsible for humoral responses and produce anti-inflammatory cytokines like IL-4 and IL-10. Following successful implantation, the immune response of the endometrial lining transitions from a cell-mediated to a humoral response. At the site of placental implantation, the prevailing TH2 immunity supersedes TH1 immunity and helps to safeguard the fetus by maintaining a balance between the two immune responses and supporting the growth and development of the placenta. However, if there is an unequal distribution of Th1 and Th2 cells and their cytokines, it can lead to implantation failure (18).

Recent studies have brought to light the crucial role of natural killer (NK) cells in pregnancy (8). The functionality of NK cells is governed by a balance between activating and inhibiting signals, which not only confer direct cytotoxic properties but also exert protective effects via cytokine production. NK cells are categorized into three different groups based on their receptor families, namely, killer immunoglobulin-like receptors, C-type lectin family (CD94/NKGs), and immunoglobulin-like transcripts (ILTs or LIRs), and their receptor repertoires vary between individuals. During the pre-ovulatory stage of the menstrual cycle, the number of NK cells is minimal, but it increases during the secretory phase as progesterone levels rise (21). In the event of pregnancy, NK cells comprise approximately 70% of all mononuclear cells in the decidua and exhibit their full range of activating receptors, including NKp44, which is frequently observed post-NK cell activation (22). Reduced levels of NKp44 have been linked to reproductive failure. It is worth noting that uterine NK cells are distinct from peripheral blood cells since they are primarily CD56brightCD16−, whereas blood NK cells are CD56dimCD16+. Uterine NK cells perform a critical function in establishing a normal pregnancy by communicating directly with invading trophoblasts (23). They exhibit cytotoxic activity to regulate trophoblast invasion and modulate the immune response locally via TH2- and TH3-type cytokines. However, they can also express classical NK cytotoxicity and alloimmune reactions, leading to the recognition of the fetus as “non-self (22).” The involvement of natural killer (NK) cell cytotoxicity in adverse pregnancy outcomes is well-established. In a study by Hadinedoushan et al. (24), NK cytotoxicity was significantly higher in recurrent spontaneous abortion patients compared to healthy controls. Similar observations were reported by Yamada et al. (25) who identified preconception NK abnormalities as a significant cause of recurrent abortions in females. Thum et al. (26) investigated predictors of successful *in vitro* fertilization outcomes and found that serum tumor necrotic factor (TNF)-alpha and interferon (IFN)-gamma levels had no relation with recurrent abortion. However, high levels of TNF-alpha and IFN-gamma were associated with elevated levels of activated NK cells, which could be a risk factor for subsequent abortion. Perricone et al. (27) suggested immunotherapy to reduce the high levels of NK cells in such patients.

For a pregnancy to be successful, it is crucial that the mother’s immune system does not attack and refuse the fetus. One of the crucial mechanisms for the success of pregnancy involves the suppression of T helper (Th) 1 cells and the upregulation of Th2 cells. This is known to be an essential process for down-regulating the cellular immune response (28, 29). Earlier studies on Th1/Th2 immune responses during pregnancy have demonstrated that both in murine models as well as human pregnancy, there is a significant inclination towards Th2-type reactions at both systemic sites (30, 31) and the fetomaternal interface (28).Treatment with immunoregulatory therapy might be able to show beneficial effects in addressing immune imbalances. The use of medications such as prednisone (PDN), cyclosporine (CsA), or hydroxychloroquine (HCQ), has been shown to suppress the secretion of Th1 cytokines, enhance the regulatory T cells’ number, and promote the development of maternal-fetal acceptance (32). Immunosuppressants such as tacrolimus have been found to be effective in the case of implantation refusal or recurrent loss of pregnancy (33, 34). Since the prevalence of *in vitro* process of fertilization (IVF) and embryo transfer (ET) has increased significantly in current times on a global scale (35) which has been accompanied by a rise in the number of women who have undergone multiple unsuccessful attempts of IVF including repeated implantation failures (RIFs), the importance of immunosuppressants cannot be ignored.

## Successful embryo implantation and immunosuppressants

Between 2 and 5 days after conception, an embryo is transplanted to the uterus through *in vitro* process of fertilization (IVF) and embryo transfer (ET). Successful implantation of the embryo into the maternal decidua, which involves building up the immunological tolerance of mother to the semi-allograft embryo, is necessary for pregnancy to occur (36). Successful implantation depends on establishing appropriate immune responses during the implantation process, which suggests that immune factors may contribute to repeated implantation failures (RIFs) following IVF/ET. The immune response involves the interplay of Th1 and Th2 cells, which are responsible for promoting either immune tolerance or rejection (37). There is a consensus that during pregnancy, there is a prevalence of immune response of T helper 2 (Th2), while an immune response of T helper 1 (Th1) is associated with refusal of the embryo (8, 38, 39). Therefore, the Th1/Th2 equilibrium model has been suggested to account for maternal-fetal immune interactions during pregnancy because critical roles in immunological responses are played by T helper cells (Th1 and Th2), which are responsible for either increasing immune acceptance or refusal (40). In general, pregnancy is characterized by a prevalence of immune response of T helper 2 (Th2), except during parturition and implantation. Conversely, an excessive immune response of T helper 1 (Th1; including TNF-α and IFN-γ) at the time of implantation is connected with miscarried implantation, early loss of pregnancy, and recurrent pregnancy loss (8, 38, 39). Women who experience RIF following assisted reproductive technology (ART) treatment have been found to have increased proportions of Th1/Th2 cell. However, treatment with immunosuppressants has been shown to enable approximately half of the women with elevated ratios of Th1/Th2 and RIF to achieve pregnancy (18). T helper subtype interactions have been suggested as one of the contributing factors to the success of in IVF pregnancy. However, it is important to note that the local and systemic roles of these interactions vary. Their clinical assessment in IVF units is subpar and so, this tends to affect which and how immunotherapy agents are used.

## Tacrolimus as immunosuppressant therapy for reproductive failures

The use of new immunosuppressive agents has significantly improved the survival rate of transplanted grafts, as demonstrated by the notable success achieved in this area (40). One of the primary immunosuppressive agents used to minimize the alloreactivity of the immune system of recipient and lessen the chance of organ refusal following allogeneic organ transplantation is tacrolimus (41). According to studies, tacrolimus can successfully reduce the allograft’s immunological rejection and enhance its longevity. This is accomplished by limiting the formation of cytotoxic T cells, the expression of IL-2 receptors, the proliferation of lymphocytes brought on by alloantigens, and the release of soluble mediators produced by T cells, such as IL2 and IFN-c (42). Although many subsets of T cells are linked to graft-versus-host disease and other immunological disorders including rheumatoid arthritis, tacrolimus has been demonstrated to successfully regulate these conditions (43, 44). Increased Th1 immune response and an elevated ratio of Th1/Th2 cell have been observed in women suffering from RIF (38), indicating the potential for an immune-suppressive agent like tacrolimus to enhance rates of implantation and outcomes of pregnancy in such individuals, particularly those with heightened Th1 immunity.

The efficacy of tacrolimus has been tested through years of research. A prospective cohort research was conducted in 2014 evaluating the clinical significance of treatment with tacrolimus in women suffering from RIF. The results were compared between a group who were treated with tacrolimus and who did not receive any treatment. Tacrolimus was administered to the treatment group beginning 2 days before the transfer of embryo and continued until the day pregnancy test was taken. Altogether, it was a period of 16 days. The findings of the research observed that the group who did not receive any treatment had 0 % rate of clinical pregnancy while the group treated with tacrolimus showed 64 % pregnancy rate. The miscarriage rate in the treatment group was found to be 6.3% while percentage of live births was 60 % (18). A 2017 study investigated whether the level of T helper 1 (Th1) cells in peripheral blood could forecast the pregnancy outcome in patients with a past of RIF following ART cycles. The patients who received the treatment were separated into three groups based on the level of Th1 cell. The findings of the research established that a negative connection exists between outcome of pregnancy and Th1 cells in peripheral blood and the proportion of Th1/Th2 cells is a prognostic pointer for the outcome of ART in patients suffering from RIF who received tacrolimus treatment (45).

A case study also reported despite prior treatments with unfractionated heparin, low-dose aspirin, intravenous immunoglobulin, and prednisolone, a patient who was treated with tacrolimus achieved a successful pregnancy after experiencing 12 consecutive miscarriages. The number of Th2 cells were reportedly increase with tacrolimus treatment and the ratio of Th1/Th2 was decreased demonstrating that miscarriage can be avoided with dominance of Th2 cells (46). By securing to the immunophilin FKBP12 (FK506 binding protein), tacrolimus creates a new complex that reduces the activity of peptidyl-prolyl isomerase. This complex, known as FKBP12-FK506, not only interacts with and inhibits calcineurin but also blocks the signal transduction of T-lymphocytes and transcription of IL-2 (47). Another 2020 clinical study determined if tacrolimus treatment for women who had experienced RIF was effective on their endometrium. The study observed that tacrolimus upregulated IL-10, LIF, and IL-17 while down regulates the proportion of IFN-γ/IL-10, IFN-γ, and IL-4 in RIF patients. In RIF patients with high ratios of Th1/Th2 cells, who had previously undergone at least three embryo transfers without success, treatment with tacrolimus resulted in a 40 % rate of implantation, 50 % rate of clinical pregnancy, and thirsty-5 % rate of live births. The study also established a noteworthy association between the intensities of IL-10 and the implantation rate, which showed a positive correlation (48).

A 2022 study investigated the alterations in the populations of Th1 and 2 cells in the peripheral blood of RIF patients who were treated with tacrolimus, delivered a live born baby, and were in the course of pregnancy. Two groups were made based whether the patients have suffered recurrent pregnancy loss (RPL). The study found that in both the groups, tacrolimus treatment suppressed the Th1 immunity, however, in the group with RIF-plus-RPL, it was observed that the percentage of Th1 cells decreased slowly after the start of the treatment (49). In RIF patients, tacrolimus was established to pointedly enhance the expression of IL-10, LIF, and IL-17 while reducing the expression of the proportion of IFN-γ/IL-10, IFN-γ, and IL-4. Moreover, the treatment resulted in a gradual decrease in the numbers of Th1 cells. Given that the group with high concentration of Th1 displayed a markedly lower ongoing rate of pregnancy and higher rate of miscarriage, it is possible that an increase in Th1 cells can cause pregnancy losses and implantation failures. As a result, it is recommended that tacrolimus treatment should not be stopped after the setting up of implantation of embryo in the mother. The safety of tacrolimus has been demonstrated during pregnancy. Furthermore, the use of tacrolimus has also been associated with inhibited fetal malformation rates, restored morphology of spiral arterial modification, and improved uterine arterial and umbilical blood flow (50, 51). Currently, topical tacrolimus is used off-label in young children, however, data on long-term safety is relatively scarce. A study by Salava et al. (52) reported a comparable safety and efficacy of topical tacrolimus (0.03 and 0.1%) and topical corticosteroids in children with AD. Similarly, a review found no evidence of increased infection risk or cancer after a 4-year follow-up in patients who used tacrolimus ointment (53). More comprehensive and randomized controlled studies with double-blind methodology are needed to establish the efficacy of tacrolimus in resolving pregnancy issues caused by immune system anomalies.

## Cyclosporin as immunosuppressant therapy for reproductive failures

Cyclosporine (CsA) was first identified as an immunosuppressant in 1976 (54). Both cell-mediated and humoral immune responses are suppressed by it (55). In order to avoid organ refusal or other autoimmune diseases including systemic lupus erythematosus and rheumatoid arthritis, it is frequently administered as an immunosuppressant (56–59). A complex is formed by cyclosporine with cyclophilin to amplify its effect and this complex in turn binds to calcineurin and hinders with the production of lymphokines including IFN-ɣ, TNF-α, and interleukin 2 as well as lymphocyte proliferation (IL-2). These are carried out by inhibiting serine–threonine protein phosphatase activity, which causes the immune system to be downregulated (60). Studies on the effectiveness of cyclosporine in enhancing RIF patients’ outcomes are few. A 2021 study examined the effects of cyclosporine on the outcomes of clinical pregnancy in women who have suffered inexplicable transfer refusal in cycles of frozen–thawed embryo transfer (FET). However, the study was not able to obtain any beneficial results for outcomes of clinical pregnancy (61).

A recent study by Cheng et al. looked at the impact of cyclosporine on outcomes of pregnancy in RIF patients. The study found that the use of cyclosporine after embryo transfer led to a significant improvement in rate of implantation, rate of clinical pregnancy, and rate of live birth among RIF patients, with no increase in the chances of pediatric or obstetric complications (62). Moreover, patients with refractory immune recurrent spontaneous abortion (RSA) who failed to respond to prior therapies with prednisone, aspirin, heparin, IVIG, and lymphocyte immunotherapy, and who were also suffering from antiphospholipid syndrome (APS) were treated with cyclosporine. Cyclosporine was effective in reducing the levels of autoantibodies, resulting in a 76.92% success rate in achieving pregnancy (63). The safety, impact and mode of action of low-dose cyclosporine in RSA patients were evaluated by Ling et al. When pregnancy test came out positive, the treatment group started taking oral cyclosporine at a dose of 100 mg/day for 30 days while the progesterone was used in the control group. Assessments of immunologic parameters were made before and after the treatment. After therapy, maternal blood’s CD3 level increased while its CD8 level decreased. Additionally, the cyclosporine group had a considerably greater live birth rate. There were no negative pregnancy outcomes or side effects (64).

Furthermore, in their trial, Azizi et al. enrolled 76 women with RPL (38 women each group treated with cyclosporine and the control group), and they investigated immunologic parameter changes in addition to pregnancy outcomes before and after the treatment. The results exhibited that after receiving cyclosporine, there was a momentous decrease in the Th1 cells’ frequency, the proportion of Th1/Th2, the expression of T-bet (a Th1-related transcription factor), and the secretion of TNF-α and IFN-ɣ, compared to pre-treatment. No significant changes were observed in the control group. Additionally, cyclosporine significantly increased the Th2 cells’ frequency, the expression of GATA-3, and the IL-10’s release. There was also a significant increase in the rate of successful childbirth in the cyclosporine group (65). Recently, Zhao et al. conducted a randomized controlled trial to scrutinize the intrauterine perfusion efficacy of cyclosporine in women with RSA who were also suffering from endometrial alloimmune dysfunction. The results showed a significantly higher rate of live births in group treated with cyclosporine in contrast to the control group. Additionally, in the second menstrual cycle, the frequency of CD57+ cells and CD56+ cells during the luteal phase o was lower in the cyclosporine group (66). So far, very little evidence is available regarding the safety of CsA, however, it is generally considered safe. Some studies have shown that CsA use can lead to increased risk of preterm birth and low birth weight, however, no congenital defects have been reported (67). CsA can cross placenta and the concentration in fetus has been shown to vary between 37 and 64% compared to maternal concentration (68). Various studies have addressed the use of Cyclosporine among patients with acute myocardial infarction. A systemic review of 6 RCTs reported no serious adverse events in all the studies (69). Similarly, in another study, the safety and efficacy of cyclosporine were shown in children treated for vernal keratoconjunctivitis (70). A case series by Leonardi et al. (71) also concluded that cyclosporine is safe in patients treated with AD. Despite ongoing research, the usefulness of cyclosporine for patients with persistent spontaneous abortion and persistent implantation refusal is currently only partially supported by high-quality data. Given the lack of robust evidence, the use of cyclosporine for these patients is not recommended and should only be used within the context of clinical trials.

## Sirolimus as immunosuppressant therapy for reproductive failures

Sirolimus, commonly called rapamycin, is an immunomodulatory agent that has been approved by the FDA for the inhibition of refusal in solid organ transplants. In addition, sirolimus has been shown to have anti-tumor effects. The immunosuppressive action of sirolimus is achieved through its inhibitory effect on the mammalian target of rapamycin (mTOR) kinase pathway, which blocks downstream co-stimulatory signals (72). The T regulatory cells’ expansion, the interception of differentiation of Th17 cell, the bocking of proliferation of T and B lymphocytes by stopping the IL-2 and IL-4’s development, and the reduction of responses due to inflammation are some of the proposed mechanisms through which sirolimus exerts its action in moderating the immune system (73, 74). Sirolimus is not a contraindication for pregnancy, according to the national transplantation pregnancy registry (NTPR), which noted that over 14,000 female transplant patients globally had previous successful pregnancies (75).

Furthermore, animal studies also supported sirolimus’ beneficial effect on gestation. A study on a mouse model of RIF showed that sirolimus was capable to increase the number of Treg cells and improve implantation rates in mice short of regulatory T cells (DEREG) (76). In a Phase II randomized clinical trial conducted by Ahmadi et al., the sirolimus’ impact on immunological abnormalities in women with RIF and a history of at least three implantation failures was evaluated. The trial found that patients with an eminent proportion of Th17/Treg who received sirolimus as treatment experienced a surge in Treg cells and a decrease in Th17 cells and the of Th17/Treg. This was connected with a higher rate of clinical pregnancy and the outcome of live birth in the treatment group in comparison to the control group that were not treated with sirolimus (77). So far, no study has evaluated the effectiveness of sirolimus in enhancing outcomes of pregnancy in women suffering from RPL. As of right now, sirolimus has been utilized in animal models of RIF and for improving the outcomes of pregnancy of women suffering from RIF in the research by Ahamdi et al. The safety of sirolimus has been reported in several small studies; however, no large study is available (78, 79). Furthermore, adverse outcome such as miscarriage low birth weight and preterm delivery but no malformities defected has been reported. Some case studies have reported that placental transfer in sirolimus takes place, however, it is in very low concentrations (80, 81). Sirolimus has been confirmed to be effective in various diseases. A study by Adams et al. reported the safety and efficacy of sirolimus in patients with complicated vascular anomalies. The most common grade 3 or more toxicities in their study were bone marrow toxicity, gastrointestinal toxicity, and metabolic toxicity (82). Similarly, another study reported mild adverse events in lymphangioleiomyomatosis patients treated with sirolimus (83). Even, in the pediatric population, sirolimus has shown safety in childhood diseases (84). Despite the lack of sufficient evidence on the efficacy of sirolimus in cases of reproductive failure, its demonstrated ability to modulate the immune system suggests that it has the prospective to be an encouraging treatment option for reproductive and fertility problems with an immunologic origin (Table 1).

**Table 1:** A summary of different immunosuppressive treatments and their effect on live birth.

Author	Type of Study	Participants	Methodology	Live birth rates	Results
Nakagawa et al. (41)	Prospective study	25 patients with repeated implantation failure (RIF) treated with tacrolimus and 17 in control.	Tacrolimus (1–3 mg) for 16 days starting 2 days before embryo transfer.	60.0%.	Clinical pregnancy rate was 64% compared to 0% in control (*p* < 0.0001). Miscarriage rate was 6.3%.
Nakagawa et al. (45)	Prospective cohort	124 women with RIF. Groups based on Th1 cell levels: <22.8 as Low; 22.8 to <28.8 as Middle, and 28.8 or greater as High group.	Tacrolimus treatment (1–3 mg/day)	35.4%.	Clinical pregnancy rates for low = 48.8%, middle = 43.9%, and high = 33.3%
Nakagawa et al. (46)	Case report	Patient with 12 prior miscarriages	Tacrolimus (2 mg/day) from 4 weeks gestation till delivery.	Successful delivery	29 weeks delivery.
Bahrami-Asl et al. (48)	Prospective study	10 RIF patients	Tacrolimus (1–3 mg/day)	35%.	Significant increase in expression of LIF, IL-10, and IL-17 in treated group. Clinical pregnancy rate = 50%.
Cheng et al. (62)	Retrospective cohort study	Cyclosporin A (CsA) group: 62, Control: 84	-	48.39% vs. 32.14%.	Clinical pregnancy rate (58.06% vs. 38.10%).
Fu (63)	Prospective study	26 RIF patients.	CsA at 80 ng/mL to 150 ng/mL.	76.92%.	Twenty patients had premature labor (34–37 weeks).
Ling et al. (64)	Cohort study	CsA group (*n* = 66) and control group (n = 20)	Oral CsA 100 mg/day for 30 days.	62.1% vs. 30.0%.	No adverse effects in the pregnant women.
Azizi et al. (65)	Retrospective cohort study	76 RPL patients.	50 mg/day oral CsA.	81.5% vs. 42.1%.	Significant decrease in Th1 frequency, Th1/Th2 ratio, interferon-γ, and tumor necrosis factor α in treatment group.
Zhao et al. (66)	Randomized control trial	The CsA group (*n* = 101) and the placebo group (n = 100).	250 mg CsA on the 3rd and 7th days after menstruation	74.26% vs. 59.00%	Lower endometrial CD56+ cell and CD57+ cell concentrations in treated group compared to placebo.
Ahmadi et al. (77)	Randomized control trial	121 patients RIF	Sirolimus (2 mg/day) 2 days prior to ET to 15 days after ET.	48.83% vs. 21.21%	Sirolimus treatment increased in Treg cells number and reduced the frequency and function of Th17 cells. Clinical pregnancy rate was 55.81% in treated group compared to 24.24% in control group.

## Anti-TNF-α therapy for reproductive failures

Anti-tumor necrosis factor-α (anti-TNF-α) drugs were first introduced in the United States in 1998 to suppress inflammation. These drugs have proven effective in treating autoimmune diseases such as rheumatoid arthritis (85). Elevated levels of TNF-α have been implicated in recurrent miscarriages. Increased TNF-α concentrations can stimulate Th1 cell-mediated immune responses, promote the production of prostaglandin E2, leading to uterine muscle contraction, trigger the activation of the blood coagulation system, and induce upregulation of oxidative stress (86). These effects can result in placental vascular thrombosis and ultimately lead to poor pregnancy outcomes. Adalimumab and Etanercept are FDA-approved anti-TNF-α agents used to treat infertility and miscarriage (85). Adalimumab is a type of recombinant immunoglobulin G1 (IgG1) while Etanercept is a dimeric fusion protein that consists of the soluble form of TNF receptor 2 and the Fc portion of human IgG1 antibody (87). A study has demonstrated that TNF-α inhibitors can be efficacious in enhancing the rates of embryo implantation and improving pregnancy outcomes in women who experience RSA (88).

According a randomized control trial, a total of 95 patients with refractory innate immune RSA received etanercept at a dose of 25 mg per week (89). The results showed that 89.47% of the patients who received etanercept delivered a healthy baby, whereas the corresponding number was only 72.04% in the placebo group. There were also significantly lower levels of TNF-α and NK cell activity during week 4 of gestation (89). Furthermore, administering Etanercept during endometrial preparation has been shown to enhance IVF outcomes in individuals with RIF (90). Furthermore, investigations into Adalimumab have uncovered a rise in the rates of successful pregnancy among women undergoing IVF. This effect is primarily attributed to a decrease in the TNF-α/IL-10 ratio, particularly during the implantation stage (91). Anti-TNF-α therapies such as Etanercept and Adalimumab may be specific and effective strategies to reduce serum TNF-α levels (an inflammatory mediator) and improve pregnancy outcomes in women with RSA or RIF. Several studies have shown that anti-TNF-α does cause negative impacts on maternal and fetal health (92, 93). However, further studies are necessary to fully evaluate the safety of anti-TNF-α during pregnancy.

## Conclusion

Many couples have to face the fetal immunological rejection. To avoid this semi-allograft rejection, maternal tolerance to fetal alloantigens is necessary. Numerous investigations on the use of immunosuppressive agents have been conducted in this area to date. While immunosuppressive treatments like tacrolimus, cyclosporine, anti-TNF-α therapy, and sirolimus have shown promise in managing complications related to reproduction and fertility, including RPL and RIF, and have already been used in clinical settings to control reproductive problems but further research is needed to identify the best candidates in addition to negative effects. By manipulating immune cells, these immunosuppressive agents have the power to regulate infertility. Despite conducting extensive research on the subject matter, we encountered a dearth of high-quality studies that could provide robust evidence to support our findings. To protect the fetus from immune responses, there is presently no single treatment strategy with high safety and effectiveness. Therefore, additional evaluation of treatment modalities in the field of reproductive failures need more rigorous clinical trials and experiments.

## Author contributions

The author confirms being the sole contributor of this work and has approved it for publication.

## Conflict of interest

The author declares that the research was conducted in the absence of any commercial or financial relationships that could be construed as a potential conflict of interest.

## Publisher’s note

All claims expressed in this article are solely those of the authors and do not necessarily represent those of their affiliated organizations, or those of the publisher, the editors and the reviewers. Any product that may be evaluated in this article, or claim that may be made by its manufacturer, is not guaranteed or endorsed by the publisher.
